# We Don’t Have a Lot of Healthy Options: Food Environment Perceptions of First-Year, Minority College Students Attending a Food Desert Campus

**DOI:** 10.3390/nu11040816

**Published:** 2019-04-11

**Authors:** Jaapna Dhillon, L. Karina Diaz Rios, Kaitlyn J. Aldaz, Natalie De La Cruz, Emily Vu, Syed Asad Asghar, Quintin Kuse, Rudy M. Ortiz

**Affiliations:** 1 School of Natural Sciences, University of California, Merced, CA 95343, USA; jdhillon5@ucmerced.edu (J.D.); kaldaz@ucmerced.edu (K.J.A.); ndelacruz2@ucmerced.edu (N.D.L.C.); evu2@ucmerced.edu (E.V.); sasghar@ucmerced.edu (S.A.A.); qkuse@ucmerced.edu (Q.K.); rortiz@ucmerced.edu (R.M.O.); 2 Division of Agriculture and Natural Resources, University of California, Merced, CA 95343, USA

**Keywords:** barriers, college, diet quality, facilitators, qualitative research

## Abstract

First-year college students are at particular risk of dietary maladaptation during their transition to adulthood. A college environment that facilitates consistent access to nutritious food is critical to ensuring dietary adequacy among students. The objective of the study was to examine perceptions of the campus food environment and its influence on the eating choices of first-year students attending a minority-serving university located in a food desert. Focus group interviews with twenty-one first-year students were conducted from November 2016 to January 2017. Students participated in 1 of 5 focus groups. Most interviewees identified as being of Hispanic/Latino or Asian/Pacific Islander origin. A grounded theory approach was applied for inductive identification of relevant concepts and deductive interpretation of patterns and relationships among themes. Themes related to the perceived food environment included adequacy (i.e., variety and quality), acceptability (i.e., familiarity and preferences), affordability, and accessibility (i.e., convenience and accommodation). Subjective norms and processes of decisional balance and agency were themes characterizing interpersonal and personal factors affecting students’ eating choices. The perceived environment appeared to closely interact with subjective norms to inform internal processes of decision-making and agency around the eating choices of first-year students attending a minority-serving university campus located in a food desert.

## 1. Introduction

The transition from adolescence to adulthood is a critical period of increased autonomy and independence [1]. For about 20 million young adults in 2015, this represented the transition to college [2]. During this transition, students establish dietary independence and become vulnerable to unfavorable changes in diet and physical activity, which can lead to malnutrition and both acute and long-lasting behavioral and health outcomes [3]. According to the most recent data, adolescents aged 14 to 18 years consume lower than the recommended amount of fruits, vegetables, and whole grains and excessive amounts of calories from added sugar, solid fats, and alcohol [4]. A recent report indicates that adolescents in the lowest quartile of fruit and vegetable intake continue to have lower intake of those foods as young adults [5]. Poor diet quality during adolescence is associated with higher risk of developing cardiometabolic disorders later in life [6]. The prevalence of obesity among young adults is the greatest among those reporting some college education. At the same time, college and postsecondary education students are disproportionally affected by food insecurity [7,8,9].

The availability and access to nutritious food (e.g., fruits and vegetables) can facilitate nutritionally-sound food choices [10,11,12,13,14,15]. Because of their expected effects on diet quality and security, research on the impact of food systems on the availability and accessibility of nutritious food in vulnerable populations has been recently emphasized [16]. Decreased diet quality due to inadequate food access can lead to hampered academic performance, likely mediated by compromised mental health [17]. The significance of the food environment on eating behavior can be particularly critical in college campuses located in food deserts [18], where students have limited access to nutritious food due to physical and financial constraints [19]. Because perceptions are considered a core determinant of health behavior [20], the role of the perceived food environment in shaping dietary choices [13] has been emphasized in the literature. However, there is a paucity of evidence on the food environment perceptions of college students attending a food desert campus and whether and how these perceptions influence eating choices. 

A food desert, as defined by the United States Department of Agriculture (USDA), is an urban area where at least 33% of its residents are located more than a mile away from a venue offering nutritious food (e.g., supermarkets) [21]. Despite being located at the heart of the most productive agricultural region in California, the university campus where this study was conducted is a food desert, i.e., about 4.5 miles from the nearest supermarket. The vast majority of students attending this campus are from underrepresented minorities and 60% come from low-income families [22]. Food insecurity, poor diet quality, higher risk of diet-related diseases, and inadequate access to health care are typically correlated with low socioeconomic status (SES) [23,24,25]. Reports indicate close to 60% of the students at this study site have experienced food insecurity, and the proportion of students reporting being food insecure widens with every year of enrollment [26]. According to the latest university census, most students living on campus are first-year students (72%) [27]. As food insecurity increases after the first year in college [28], first-year students represent a key target study population for early prevention of food insecurity and related acute and persistent dietary maladaptation.

The purpose of the present study was to examine perceptions on the campus food environment, the personal and interpersonal factors that interact with the perceived food environment, and their collective influence on the eating choices of first-year students attending a university located in a food desert. The identification of these factors will broaden the knowledge base in the area, providing the basis to explore their relevance in shaping eating choices in future qualitative studies. Ideas to improve the campus food environment were also explored.

## 2. Methods

**Figure 1:**
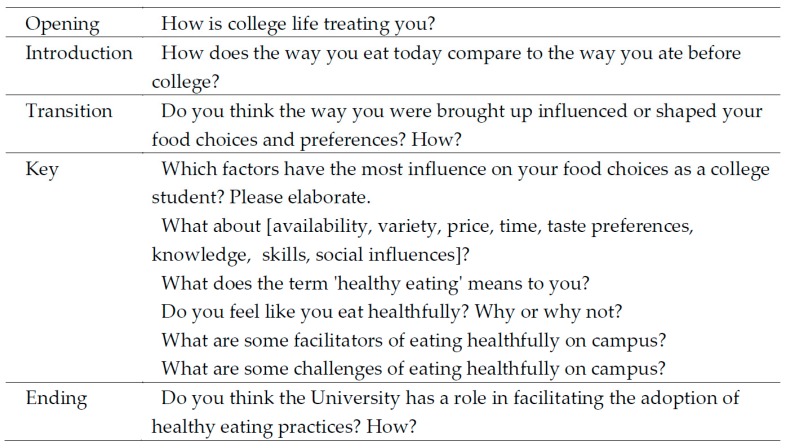
Question guide for the focus group interviews of first-year college students.

Potential participants for focus group interviews were recruited from a pool of first-year college students participating in a parent clinical study (Clinical trials ID: NCT03084003). The objective of the parent study was to evaluate the effects of 8 weeks of different snacking options on glucoregulatory and cardiometabolic profiles of first-year college students [29]. Participants for the parent study were recruited via advertisements. Students were included if they were 18–19 years old and were newly enrolled, first-year college students living on campus. Participation in the focus groups was voluntary and was open to all participants in the parent study (*n* = 73). Twenty-one students consented to participate in focus group interviews, while others did not show an interest in participation. Students were monetarily compensated for participating in the parent study but did not receive additional compensation for participating in the focus groups. This study was approved by the Institutional Review Board of University of California, Merced. Signed written consent was obtained from all participants prior to the focus group sessions, and the data were kept confidential. A semistructured question guide was developed according to recommended methodology [30] to explore factors related to students’ food choices and eating behavior in college, transition from home eating behaviors, perceived food environment, and barriers and facilitators to make nutritious choices. Questions were designed to probe constructs in the social cognitive theory (i.e., reciprocal determinism) [31] and the social ecological model [32]. Introductory and transition questions established the tone of the discussion and led to an in-depth discussion regarding the phenomenon of interest (i.e., eating choices and dietary behavior influences of first-year college students living in a low food-access campus environment). Closing questions explored students’ opinions on solutions to improve the food environment on campus. The question guide was tested with a pilot focus group of first-year students meeting the inclusion criteria of the study (*n* = 4, focus group 1), and the data were later included in the final analysis because no substantial changes to the guide were made. The general question guide is depicted in Figure 1.

Data were collected from November 2016 to January 2017. Focus groups were facilitated by a trained moderator and an assistant moderator (i.e., observer), who audio-recorded the session and took notes on nonverbal cues. The moderator led the discussion following the question guide, probing for in-depth information as needed. All focus group discussions were audio-recorded with permission from the participants. Audio-recordings were transcribed verbatim in Microsoft Word by trained scribes. Data were transferred to and organized in Microsoft Excel for analysis.

Data underwent a first round of preliminary coding done independently by 4 researchers under the supervision of an expert researcher. A codebook was developed in Microsoft Excel based on this preliminary analysis by identifying concepts from available literature [14,33] on the perceived environment and the health belief model (perceived barriers) [20], theory for planned behavior (subjective norms) [34], and the expectancy-value theory (decisional balance) [35]. A second round of coding using the codebook was done independently by 2 trained researchers to ensure reliability of data interpretations. Quotes were examined for recurrent patterns across the data set, assigned codes, and grouped into categories [36]. Similar codes were further divided into subcategories. Disagreements were resolved through further discussion with a third analyst and included contextualization based on theory and relevant literature. Codes were semiquantified to determine density and representativeness across focus groups and participants [37]. A grounded theory approach was applied for inductive identification of relevant concepts and deductive interpretation of patterns and relationships among themes and subthemes, seeking internal homogeneity and external heterogeneity [38]. A conceptual diagram was conceived to describe relationships among main themes and subthemes [39]. A negative case analysis was conducted for quotes that did not support emerging patterns [40]. Thematic saturation occurred at the third focus group (13 participants). Two additional focus groups were conducted (8 participants) to corroborate saturation and further probe salient themes. 

For descriptive purposes, 2015 Healthy Eating Index (HEI-2015) scores [41] were computed from 2 24-hour food recalls spaced a week apart in the students’ freshman year using a validated, automated, self-administered 24-h dietary assessment tool (ASA24) [42]. HEI 2015 scores were calculated using the per-person simple HEI scoring algorithm [43]. Sex differences were explored with the Kruskal–Wallis test. Significance was set at alpha ≤ 0.05.

## 3. Results

A total of 5 focus groups (*n* = 21) were conducted, lasting an average of 60 to 75 min. The majority (90.5%) of the participants were from an ethnic/racial minority, with the dominant groups being of Hispanic/Latino and Asian/Pacific Islander origin (Table 1). 

According to HEI-2015 scores, participants had low fruit, vegetable, whole grain, and dairy intake, and high sodium intake. Male participants had a significantly lower fruit (total and whole) and higher sodium intake than females (*P* < 0.05, Table 2).

**Figure 2:**
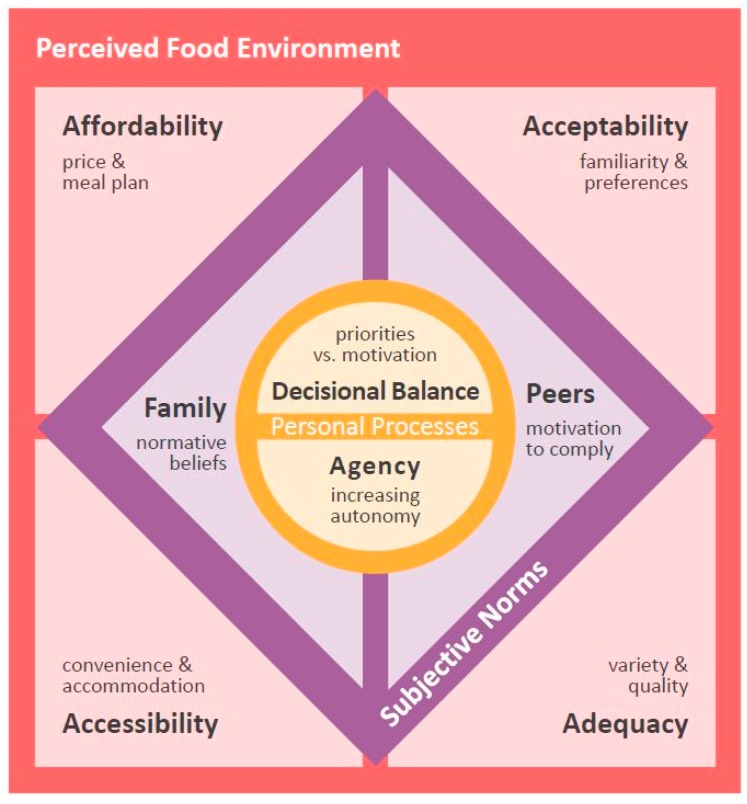
Schematic representation of the relationship among main themes, conceived upon an inductive, grounded theory analysis of focus groups with first-year college students. Personal processes of decisional balance and agency interact with four dimensions of the perceived food environment— affordability, acceptability, accessibility, and adequacy, —and with subjective norms—normative beliefs from family and motivation to comply with peers—to influence the food choices of students attending a food desert campus.

Six main themes were identified, four of which were related to the perceived food environment on campus: (1) Affordability, (2) acceptability, (3) accessibility, and (4) adequacy. Two additional themes were about the influence on eating choices of interpersonal and personal processes: (1) Subjective norms derived from family and peers, and (2) personal processes of decisional balance and agency. Solutions to improve their food environment and facilitate favorable eating behaviors were offered by participants. Figure 2 depicts a conceptual diagram devised to explain relationships among the main themes. Additional representative quotes for each theme are listed in Table S1.

### 3.1. Perceived Affordability

The cost of foods on campus was consistently and most frequently brought up as having a major influence on eating choices. Participants perceived nutritious options as more expensive than less nutritious options, which often led them to opt for the latter:

“One of my major deciding factors when I buy food is how much the cost is, and the cost is usually lower with the unhealthy food”

(P19, G5)

“I would eat more fruits, so much more often if it wasn’t so expensive; but, since it’s so expensive I always get like burgers, which are cheaper.”

(P12, G3)

Some participants considered their meal plan as an advantage to secure resources for food:

“[If I didn’t have a meal plan], I would just spend the money that was dedicated for my food, I would probably spend it recklessly. So, I think it does help that I’m only able to spend that money on food on campus.”

(P2, G1)

However, the meal plan was also perceived as restrictive for not allowing purchases from most outside food vendors, including some of the few located on campus:

“I only have [meal plan] dollars instead of real money; so, I can’t really do anything about it and get food at the vendors on campus.”

(P10, G3)

Overall, the limited affordability of nutritious options was consistently considered a barrier to healthy eating. Students’ recommended solutions to minimize this barrier included allowing the use of their meal plan for purchasing food from third-party vendors on campus, making cheaper fruits and vegetables available on campus, and living off-campus after their freshman year for better access (e.g., proximity, transportation) to a greater variety of food options in terms of nutritional quality and price.

### 3.2. Perceived Acceptability

Students’ food expectations appeared to be mostly related to their taste preferences and familiarity with available food. These expectations seemed to influence their perceptions on the quality of food on campus, which in turn led them to adjust their eating behavior. For some, the perceived negative organoleptic qualities of food were largely attributed to food preparation seen as inferior to that of familiar food:

“…when I eat the chicken here, it isn’t like the chicken back home—it doesn’t really taste like chicken to me. But, I usually don’t eat at the [campus dining center]. If I do, I just get—if I’m desperate only and usually it’s not appetizing—I get the peanut butter sandwich”

(P5, G2)

“I don’t know if they make [the eggs] the day before or what, but, I wish it would taste like they were just made.”

(P21, G5)

A few students contended familiar foods were not as available on campus:

“My family is White; so, the food that I’m used to eating is more plain, and the food that they have here has a lot of ethnic foods and I don’t know what it is. So, I don’t get it because I don’t want to waste money on it if I don’t like it.”

(P10, G3)

Broadening the variety of food options and outlets on campus was among the ideas students offered to improve the campus food environment and, thus, their eating behavior. They also recommended a number of operational changes on the campus dining center, including posting menus in advance, making nutritional information of menu items readily available, and improving and varying food preparation techniques.

### 3.3. Perceived Accessibility

Factors related to the convenience of accessing food were saliently expressed as having a significant influence on eating behavior. Students indicated proximity to campus food outlets greatly influenced their eating choices. Proximity to places that offer energy-dense options and inconvenient location and schedule of fresh produce outlets were expressed as barriers to healthy eating:

“My first semester, it was bad—I eat all day, especially since I live in [a residence hall close to the dining center], I can just walk up to the cafeteria whenever I want. [The] first semester I probably ate more burgers and burritos than I should have.”

(P14, G4)

“Occasionally there is the fresh fruit produce truck; but, they are only here on Wednesdays and occasionally it is hard to go up the hill and go there to get fruit.”

(P8, G2)

Limited transportation options to off-campus grocery stores and food joints was also perceived as a barrier to accessing food, especially under the competing priorities imposed by academic demands:

“The bus takes too long; so, it cuts my day off and it cuts time off from studying and class—like, I would have to miss class. There’s no point, I’m literally just on campus and [the campus dining center] is the main food access; so, I just stick to that.”

(P20, G5)

Along with grocery shopping limitations, inconvenient access to cooking facilities was brought up as a factor affecting eating choices:

“If I had an option to cook and get groceries easily, I don’t think I would rely on the [meal] plan so much.”

(P1, G1)

“Reservations [for the campus kitchens] are always a hassle. So, there are days when I…don’t want to go to the [campus dining center]; but, knowing that you have to make [kitchen] reservations, just make you want to go to the [campus dining center].”

(P19, G5)

Several students indicated they often rely on food provided by their family to have enough and familiar food available. They reported bringing familiar foods from home, especially items that would conveniently withstand storage:

“As much as I can, I always try to bring stuff back [when I go home]. But, I never bring vegetables or fruits. I bring anything that’s frozen, so that it can survive the trip and doesn’t get nasty.”

(P20, G5)

“I live an hour away, and my mom usually will freeze things and I’ll put it in the freezer [sic].”

(P13, G3)

“I’m Nigerian, so I bring a lot of cultural food because I miss home. I also stock up on cereal like Costco bulk and mini snacks, like fruit snacks.”

(P17, G4)

Potential solutions proposed by students to make accessing nutritious food more convenient included expanding the frequency and hours of operation and improving the location of produce outlets on campus, as well as better transportation options to off-campus food retailers.

### 3.4. Perceived Adequacy

According to participants’ input, their food choices were generally influenced by on-campus availability of adequate options. As first-year students, most relied on the meal plan offered by the university to cover their dietary needs and indicated that the majority of their meals were consumed at the campus dining center. In general, they characterized the food and food establishments on campus as limited in number and lacking variety:

“There’s only like 3 different places to eat around here [on-campus].”

(P13, G3)

“I eat whatever is available to me—like, whatever is at the [campus dining center].”

(P8, G2)

“I pretty much eat anything at the [campus dining center], even [if] I don’t like it, because I don’t really have a choice.”

(P20, G5)

Most participants judged the food available on campus as nutritionally inadequate, with nutritious food options being scarce:

“I feel like we don’t have a lot of healthy options [on-campus].”

(P2, G1)

“The only thing they have of fruit [at the campus convenience store] are the cups; and there really isn’t anything healthy.”

(P7, G2)

Conversely, a few students seemed content with the food options available on campus:

“The food overall here is a lot healthier than I had back in my hometown.”

(P1, G1)

“They always have new things every day [at the campus dining center], and I’m willing to try new things.”

(P11, G3)

### 3.5. Subjective Norms

Norms that governed their eating behavior before college, along with new normative beliefs acquired by being immersed in a new social environment, appeared to mediate students’ perception of and interaction with their food environment. In terms of pre-college influences, students indicated health history and eating practices at home were ways their family influenced their eating choices:

“We have like a pretty bad history with health. Something shifted in my parents’ way of thinking and then they went super healthy. I’ve been trying to move towards the healthier side.”

(P1, G1)

“[My siblings and I] we all like to eat a lot, and our parents never told us we should slow down. They always told us ‘you need to grow and eat a lot.’ So, it kind of influenced what I eat here. Like, when I first came here I was like ‘oh, look at these options,’ right? And I was like eating like $30 a day for like the first 2 or 3 weeks. After that, I was realizing that I should probably slow down on this.”

(P17, G4)

Other comments alluded to the role of fellow students in shaping new normative beliefs around their food choices:

“My roommate tried to get me to eat healthier, in a way, by having me get like cereal from the [campus convenience store] and stuff.”

(P20, G5)

“I remember there was time when [I wanted to] eat healthy and just eat salad every day. But, my friends said that salad was more expensive; so, I agreed. So, it’s cheaper to just get pizza. She kind of influenced me to not get salads because it’s more expensive.”

(P12, G3)

### 3.6. Personal Processes

Along with environmental and normative factors, personal decision-making processes requiring self-evaluation and introspection were also raised by participants when discussing eating choices. Students often described going through an internal conflict of choice, particularly when deciding between more and less nutritious food options:

“You have a healthy option and a non-healthy option, like a burger and fries or veggies. And you must decide if you want to eat healthy or not—the internal conflict of being healthy.”

(P14, G4)

Participants mentioned competing priorities with respect to time, money, and academic demands importantly weighing in, sometimes to the detriment of their eating choices:

“We have to find something to eat in a certain amount of time. It doesn’t matter if you like it or not, you kind of just have to eat it.”

(P13, G3)

“I was always more focused on saving money [than eating healthy].”

(P4, G1)

“[Healthy eating] starts becoming less important around finals.”

(P2, G1)

Lack of motivation to comply with the expectations of maintaining a healthy lifestyle was also expressed:

“I would get milk and food and stuff [from a grocery store]. But, it’s too much work and I don’t think it’s important enough.”

(P10, G3)

Notably, some comments alluded to a conflict arising when pondering options. This was compounded by an awareness of increased autonomy, which required students to assert agency over their diet:

“[It was] my mom who always was like ‘here you go, eat this.’ Here, I have all these options. And I’m free to choose. And sometimes I don’t want to choose. And sometimes I do want to choose. So, it’s like I didn’t want to have that responsibility—to buy it, cook it, and choose it.”

(P15, G4)

Taking better advantage of available resources (e.g., making kitchen reservations), developing and/or deploying skills (e.g., portion control, mindful eating), as well as earning more money and living off-campus were some ideas students mentioned they could pursue to improve their diets.

## 4. Discussion

This qualitative inquiry revealed four aspects of the perceived food environment: (1) Affordability, (2) acceptability, (3) accessibility, and (4) adequacy. These four aspects appear to influence the food choices and eating behavior of first-year minority students attending a university campus, located in a USDA-defined food desert. These aspects seem to closely interact with subjective norms from family and peers to inform students’ internal processes of decision-making (i.e., decisional balance) and agency. These findings illustrate how multiple factors at several levels of influence interact to inform eating choices by preventing the continuation and adoption of desirable eating behaviors and/or reinforcing maladaptive dietary practices. Findings from this study echo the literature on the role of the physical, economic, and sociocultural environment to affect the ability to secure nutritious food [13,14,16,44,45], and notably, it is the first to extend the knowledge on how this phenomenon is expressed in first-year college students exposed to a food desert, an environment deprived of a consistent variety of nutritious options.

Affordability of available options was a major factor affecting students’ food choices. The perceived higher price of nutritious food on campus compared to food of poor nutritional value was recurrently mentioned as a deterrent to choose the former. Economic factors are main drivers of food choices for both low-income populations and college students [33,46,47,48,49]. Given the socioeconomic background of this study’s informants, economic factors were expectedly central when discussing their food choices. The majority of the students in this study were from low-income backgrounds. The cost of attendance for these students living on campus is 27% to 34% higher than that for those living off-campus, mostly due to higher cost of housing [50]; most first-year students live on campus (72%) [27]. Although a significant proportion of students in this campus are eligible for and do receive financial aid, their disposable income is seemingly low. In fact, recent reports [26] indicate the majority of the students attending this university struggle to cover their basic needs, especially when it comes to having consistent access to nutritious food. To alleviate these disparities, the university has appointed a taskforce of students, staff, and academics to execute a plan [26] that includes establishing and expanding food distribution programs (e.g., food pantries, food banks, electronic meal donations), supporting infrastructure (e.g., meal preparation facilities, transportation), financial and nutrition education, and guidance on food assistance applications. Monitoring the effectiveness and relevance of these activities is paramount to identifying best practices and areas of improvement. Ultimately attaining a campus food environment that allows for adequate and consistent access to nutritious food is essential, especially among students at the greatest risk of malnutrition due to limited access to resources.

As emphasized by students in this study, a meal plan program can help to mitigate some of the food access challenges faced by first-year students living on campus. Having a meal plan can mean regular access to convenient meals [51]. In addition, and according to some students, having a budget committed to food may help to lessen the possibility of competing spending priorities to affect their ability to secure the food they needed in a given period. Students also revealed disadvantages to the meal plan, including poor acceptability of the food offered at approved venues and its restricted use to only designated dining locations (and not outside food or produce trucks) on campus. Internal reports indicate students have a choice between one of three meal plans, two of which are recommended for students who consume less than 3 meals per day. Thus, these meal plans may be insufficient to meet their dietary needs, especially considering all meal options offered are à la carte. Given their socioeconomic status and in an effort to maximize their food budget, these students may choose the limited meal plans and make food choices based on taste preferences and convenience rather than nutritional value, as expressed by many in this study. Access to and greater consumption of food of low nutritional quality as related to meal plans has been reported by others [51]. In this study, students’ HEI-2015 scores corroborate poor alignment with dietary recommendations [52]. Incentivizing the consumption of nutritious food by making it more ubiquitous, affordable, and appealing has been proposed to prevent dietary maladaptation in college students [45]. Some evidence suggests that eating behavior worsens with each semester in college [53] and that first-year students living on campus may be more responsive to interventions that incentivize nutritious food purchases than older students and students living off-campus [54]. 

Aspects of the physical environment, such as availability and accessibility, are expected to be particularly restricting to the food choices of those living in food deserts [11,14]. Availability of a variety of nutritious options is the dimension of the perceived food environment most consistently associated with positive dietary outcomes [14,33]. Mixed results are reported in the literature for the accessibility dimension, with some evidence indicating proximity to nutritious food outlets is secondary to the quality, variety, and price of available food [14,48]. However, most of available evidence is not specific to college settings. Moreover, in this inquiry, low variety and poor quality of available options on campus did appear to inform student food choices in favor of a less nutritious diet but not independently of and as prominently as price. For instance, although quality and variety were valued attributes, cost was described as a decisive factor in choosing one food over another. At the time this study was conducted, food outlets on campus included the campus dining center, one cold-food cafeteria, a convenience store, a few alternating food trucks from local restaurants, one produce truck visiting campus once a week, and vending machines. Although a systematic environmental mapping of food outlets to triangulate student perceptions was beyond the scope of this study, it was apparent that consistent access to affordable nutritious food was questionable, at best, in this university campus. 

Unlike reports from noncollege populations [14], convenient access to nutritious food was also deemed an important determinant of eating choices by this study’s informants. Convenience was mostly expressed as the time required to acquire and prepare food, and it was regarded in terms of proximity to food outlets and access to kitchen facilities. Based on the input from this study’s informants and similar to what others have reported [45,55], the combination of academic demands and lack of access to consistent transportation appears to be a circumstance unique to college students that makes convenience a major barrier to accessing food that affect the quality of their food choices.

Relying on food provided by family was a coping strategy to obtain desired food. In the case of this study, this process is assumed to be facilitated by geography, as the majority of students come from local communities, which allow them to visit home often. Meals provided by family seemed to have the key advantage of being convenient, acceptable (i.e., familiarity and taste preferences), and free. Family food provision has been associated with greater consumption of fruits and vegetables in European college populations [56,57]. However, evidence on the prevalence of this practice and its effects on the diet quality of college students in the US is sparse. Along with the tangible value of food provisions, sharing meals at a distance may also represent a means for preserving family connections during the transition to independence of members leaving for college. Future research may explore the significance of using food to maintain such connections, especially during the developmental milestone that is the transition to adulthood and its potential consequences on the physical and emotional health of the students and their families.

Beyond food provisions, students’ insight suggests that norms and beliefs acquired at home and new normative beliefs shaped by fellow students influenced the way they interact with their new food environment. On one hand, the quality of available food on campus was judged against the perceived quality of meals consumed before college. On the other hand, food choices were often made after views were expressed by fellow students. The way these normative beliefs interact to inform the process of making food decisions may depend on the motivation to align with norms imposed by family and peers [45,58]. In turn, motivation to comply with norms can be influenced by the intrinsic, extrinsic, and cost–opportunity value ascribed to acting on them [35,59]. Increasing the cost–opportunity value of nutritious options through a food environment that provides for affordable and consistent variety of such options may reinforce and/or help to establish normative beliefs that elicit sound dietary choices, which could be carried onto life after college.

To support the translation into action and the sustainability of positive normative beliefs acquired or reinforced in college, developing relevant skills is necessary [34,60,61]. In this inquiry, students consistently alluded to their agency at selecting and preparing food, under the circumstances of their new environment, as a determining factor for their eating choices. Comments from informants suggested that for many first-year students, a newly acquired independence and resulting increased autonomy can be a burden if skills needed to successfully navigate their new physical, economic, and sociocultural environment are underdeveloped or absent. In fact, student informants in this study consistently suggested that education-related solutions would improve the way they navigate their food environment and thus improve their eating choices. Interventions targeting nutrition knowledge and self-efficacy have been successful at improving eating behavior among college populations in the short term [62], especially when combined with environmental facilitators [33,45,63,64]. However, research on solutions that allow for sustained, long-term changes is needed. Actively offering nutrition education resources to students entering college appears to be a sensible way to mitigate barriers and amplify the impact of environmental and interpersonal facilitators to sustain a healthy diet. However, for educational interventions to be effective, they must be participatory, and framed on theory and evidence-based behavior change methods [65].

Findings from this study need to be pondered in light of its strengths and limitations. A key strength of this study is the ethnic makeup of the student informants, the majority of whom were from groups traditionally underrepresented in research [66]. Given the increasing incidence of food insecurity [28] and diet-related cardiometabolic disorders among ethnic/racial minority groups [25], studying these groups advances the understanding of this phenomenon for more inclusive practice and policy [67]. Limitations pertain to the bias risk inherent to the qualitative design [68] and informants being recruited from a nutrition study, which may indicate their interest in nutrition and diet beyond that of those who did not participate. To mitigate these risks, efforts to ensure trustworthiness were made, including the application of well-established methods by experienced and trained researchers, having analysts from the population of interest (i.e., undergraduate students), and considering theory and evidence in the interpretation of the results. Additionally, student informants were encouraged to be as open and thorough as possible. Moreover, all interviews were conducted by the same trained moderator, who applied recommended techniques to create a safe, nonjudgmental atmosphere. 

## 5. Conclusions

The focus of this study was on first-year college students because of the potential for early prevention of eating behavior maladaptation, such as skipping breakfast, increased snacking, and low intake of fruits and vegetables [69] which can be tracked forth into adulthood [5] and increase the risk of weight gain and other cardiometabolic disorders. Adequacy (i.e., variety and quality), acceptability (i.e., familiarity and preferences), affordability, and accessibility (i.e., convenience and accommodation) are the aspects of the perceived environment that appeared to closely interact with subjective norms to inform internal processes of decision-making (i.e., decisional balance) and agency around the eating choices of first-year students. Perceptions are a core determinant of health behavior; thus, characterizing the perceived food environment is necessary to understand its influence on eating choices. This is particularly critical for those exposed to a nutritionally deprived food environment and simultaneously transitioning to independence. Future research should explore how these factors of the perceived environment are expressed in representative samples of college populations attending food desert campuses, and examine how student perceptions on the food environment manifest into action and transform as they become more acquainted to life in college. Although the influence of emotional health on food choices was discussed by only a minority of students in this inquiry, it deserves further examination as it has been found relevant by others [70,71]. Future robust empirical research should explore whether or not these findings hold among college students from a variety of demographic, socioeconomic, cultural, academic, and living conditions. 

## Figures and Tables

**Table 1:** Characteristics of focus group participants, i.e., first-year college students attending a minority-serving university located in a food desert.

Characteristics	Males (*n* = 9)	Females (*n* = 12)
Age (years)	18.2 ± 0.4	18 ± 0
BMI (kg/m^2^)	24.5 ± 3.8	25.2 ± 3.7
Race/Ethnicity, *n* (%)		
African American	1 (11.1%)	0 (0%)
Asian/Pacific Islander	3 (33.3%)	4 (33.3%)
Caucasian White	2 (22.2%)	1 (8.3%)
Hispanic	3 (33.3%)	7 (58.3%)

Values are means ±  SDs or *n* (%).

**Table 2:** 2015 Healthy Eating Index (HEI-2015) total and component scores for focus group participants, i.e., first-year college students attending a minority-serving university located in a food desert.

HEI-2015 Dietary Component	Males (*n* = 9)	Females (*n* = 11 *)
Total fruits (5)	1.2 ± 1.2	2.8 ± 1.8 **
Whole fruits (5)	1.3 ± 1.7	3.6 ± 1.5 **
Total vegetables (5)	3.3 ± 1.2	3.2 ± 1.3
Greens and beans (5)	3.1 ± 2.4	2.3 ± 2.3
Whole grains (10)	3.5 ± 2.4	3 ± 3.1
Dairy (10)	4.7 ± 2.4	4.4 ± 2.5
Total protein foods (5)	4.3 ± 1.7	4.2 ± 1.4
Seafood and Plant Proteins (5)	3 ± 2.5	4.1 ± 1.8
Fatty acids (10)	5.8 ± 3.3	6.8 ± 3.5
Refined grains (10)	4.1 ± 3.3	5.4 ± 3.4
Sodium (10)	1.8 ± 1.9	4.5 ± 2.7 **
Added sugars (10)	9.1 ± 0.9	7.7 ± 2.9
Saturated fats (10)	6.2 ± 2.3	5.6 ± 3.3
Total HEI 2015 score (100)	51.5 ± 10.9	57.6 ± 14.5

Values are means ± SDs. Numbers in parenthesis are maximum possible score values. * One participant did not complete 24-hour recalls. **, *P* < 0.05 using Kruskal–Wallis test. HEI 2015 scores were calculated using the per-person simple HEI scoring algorithm [43].

## Supplementary Materials

The following are available online at https://www.mdpi.com/2072-6643/11/4/816/s1, Table S1: Additional representative quotes from focus group participants within thematic categories.
