# Supplementary material for: We Don’t Have a Lot of Healthy Options: Food Environment Perceptions of First-Year, Minority College Students Attending a Food Desert Campus
Source: Nutrients. 2019 Apr 11;11(4):816. doi: 10.3390/nu11040816 (PMC6520682; doi:10.3390/nu11040816)
Supplement: Supplementary file 1 [file nutrients-11-00816-s001.pdf]

Table S1: Additional representative quotes from focus group participants within thematic categories

Themes	Selected Quotes
Perceived adequacy—variety and quality	
Variety	"I wish there was more, like I eat an apple a day, lettuce from the salad bar, carrots, that's like two main things." (P21, G5)<br>"It's always the same thing every day, over and over." (P21, G5)<br>"Basically, the same thing. I eat at the [campus dining center] every day, it's basically like I eat what's there which is either quesadillas or fries." (P19, G5)<br>"This is my option for the day, so I will eat it." (P14, G4)
Quality	"Yeah, like my parent's cook too but they would incorporate more vegetables. Even if [my mom] put it in rice or salad with home cooked meals which I feel like I don't get any more at the [campus dinning center]." (P5, G2)<br>"I think it's bad too. I don't think there's enough healthier options." (P3, G1)
Solutions	"Knowing what they have at the different food trucks or at the [campus dining center] because sometimes you don't know what they have. And, you feel lazy and don't want to go look but [the dining center] having something healthy that day, will make me want to go." (P16, G4)<br>"They do have vegetables but maybe if they had different vegetables and different ways they are prepared, like sauce with one and no sauce with another." (P2, G2)

	"They should have more vegetarian meals." (P2, G1)<br>"Nutritional facts, like what are the ingredients that went into this food? Like, did we use, uh soy? Or just knowing what went into it." (P14, G4)<br>"If they were to have more of a variety of food." (P20, G5)
Perceived acceptability—familiarity and preferences	
Familiarity	"Honestly, I'm a really picky eater with anything and I'm used to home cooked Mexican meals and the [campus dining center] does not even have any real, good Mexican food." (P13, G3)
Preferences	"I tried everything that I possibly could, like I even tried food from [when the campus dining center has 'Meatless Mondays'] and everything, but sometimes it was cold and sometimes it wasn't. Most of it, since it's repeated, even if I like it I just get thrown off." (P20, G5)<br>"…the tofu they serve, they don't do it that well cause basically it just seems like they just buy it and cut it into pieces and it's not how tofu is supposed to be like. People do eat it like that, but there are just other ways." (P21, G5)
Perceived affordability of available food	
Campus food	"Also, here at the [campus dinning center] it is very hard to get fruit and vegetables than at home because my mom usually brings it from the groceries, but here you have to buy it yourself and is more expensive then the meals." (P16, G4)

	“I do [buy cheaper food] the same thing. The chicken strips and the fries’ cost like $4 and the strawberries that I want to take to class is like $5.” (P20, G5)
Meal plan	“Most of us, or me, I’m not financially stable so my parents are really poor, and I’m just stuck with my meal plan.” (P20, G5)
Solutions	“Letting us use our meal plan for that [other food purchases] then I would definitely buy it [fruits and vegetables] from there.” (P20, G5)<br>"I feel like if fruits and vegetables were cheaper I’d be eating more of them especially now I feel more deprived of fruits and vegetables and the only substitute is the fries.” (P17, G4)<br>“Yeah, I’m actually thinking between staying on campus or finding a place off campus. I don’t know yet we’ll see. I want to be able to cook my own foods and like it’ll be cheaper, way cheaper [off-campus].” (P12, G3)
Perceived accessibility—convenience and accommodation	
Convenience-proximity	“The [campus convenience store] is right where I live and that’s where all the junk food is so that’s what I get.” (P19, G5)
Convenience-transportation	“I think it’s the case of limited transportations because we have all these stores, but we don’t usually want to go out to get it and we don’t have cars to go and get them.” (P15, G4)<br>“Waiting isn’t my issue but the times the bus comes it’s like every hour and back in Los Angeles it was like 15 minutes for every bus to pass by.” (P19, G5)

Convenience-cooking	"It's just a hassle to just go and buy everything and cooking it since we don't have an easy access to a kitchen." (P12, G3)<br>"It's really hard for me to go off campus to go buy stuff to cook so that's what I guess has been eating me and keeping me from going on the healthy side." (P20, G5)
Accommodation-family support	"Every time I go home, and I only go home during the long breaks, I bring like a boat load of food from home, like you should see the day I come back from break. I bring like a wall of food and by the end of the semester it is empty it is sad waking up." (P17, G4)<br>"I usually bring a container of my mom's food cooking and in a week its gone because my roommates love it too so it's like goodbye food. It's like a Vietnamese thing where we rice and meat and eggs together as like a soup and we eat it together." (P15, G4)<br>"I don't bring meals because my parents want me to finish my [meal plan dollars] so I must eat at the [campus dinning center]. I also bring cereal and have a whole snack container, but I bring my own milk because I can't drink the milk here. [I drink] lactose free milk, so I must ration that out for the time that I am here." (P16, G4)<br>"My mom she would make soup for me, but that will only last like three days so it's not a lot." (P18, G4)<br>"I just bring like 3 tamales with cheese and jalapenos and a bag of carrots and celery and that's it. And only once a month I go back." (P21, G3)

Solutions	"Let's say if they had the fruit stand over there [by classrooms/ dorms], I would most likely buy fruit from over there rather than walking all the way to the other end of campus." (P19, G5)<br>"I think it would be cool to have the [pop-up, mobile produce cart] on campus more often because they only come once a week." (P11, G3)<br>"It would also help if [the campus dinning center] extended their hours because last semester I had class during the time they would be selling, so when I did have my [meal plan dollars] there was still no point for me to go and I would have to ask my roommate, but my roommate would have classes too or labs. So yeah, that would help if they would extend their hours." (P15, G4)<br>"Transportation [would help]. If I had a car I would be going to the grocery store and stocking up there instead of just going to the [campus dining center] because every time that I go it's because of convenience and I would rather go to the grocery store and off campus [eateries] to eat something different." (P17, G4)
Subjective norms—family and peers	
Family-normative beliefs	"But they [parents] were never picky with food, so I was brought up trying different things, so I'm not picky and I'll pretty much stuff myself with whatever I see, so I think that's what added to my weight gain here." (P20, G5)<br>"For me, I was brought up on having to finish all the food that was on my plate." (P19, G5)

Family-motivation to comply	“My freshmen year, my brother got too much cholesterol and so [my mother] started incorporating healthier foods in our daily food and that’s when I started eating healthier I guess.” (P19, G5)
Peers-motivation to comply	“Sometimes my friends would want to go eat at night, but I’m already too full, so I want to go hang out, but I feel awkward not eating and just sitting there, so I would get something to eat too but that just makes me feel bloated.” (P18, G4)<br>“I just go eat whatever I want to eat.” (P7, G2)<br>“Here I just eat whatever I want to eat, but I don’t care what they [friends] are eating.” (P15, G4)<br>“My roommates and friends are all close, but we are independent so when someone says they are going to eat, we say bye and go eat by ourselves.” (P14, G4)<br>“I influenced my friend to go to the gym. Sometimes he influences me.” (P3, G1)
Personal processes—decisional balance and agency	
Decisional balance-priorities	“I have back to back classes, so sometimes I don’t have time to eat or I eat late or between classes or I just eat in class.” (P18, G4)<br>“My mentality is that you must eat. So, I don’t, think it should matter how much it [costs] because you must eat anyways.” (P15, G4)<br>“You have to be flexible and sometime take shortcuts.” (P1, G1)
Decisional balance-motivation	“I stop myself from eating at a certain time, so I don’t feel weird but it’s hard to do that because I’m used to snacking so it’s difficult.” (P18, G4)

	"I did get unhealthy and I gained 14 pounds." (P20, G5)<br>"Now I eat when I'm bored and there is nothing to do so I just eat." (P14, G4)<br>"I love chocolate, and when I'm stressed out I feel the need to snack and all I have are boxes of chocolate." (P3. G4)<br>"It's hard to eat regularly because I'm either starving or not hungry at all so it's hard to find a balance." (P10, G3)
Agency	"Freedom of choice [is challenging]. So it's your decision to eat what you want [at campus] but at home it's a certain food my mom brings, so I eat what I want." (P16, G4)<br>"There are more options [at the campus dining center] that I get, and I lean more towards the unhealthy side I guess and all of the stress eating [on campus]." (P19, G5)<br>"Sometimes, I do want to get fruit and even though I'm not a big fan of melon, sometimes I'm too lazy to come back for another trip just to get fruit" (P20, G5)
Solutions	"…make a reservation for the [on campus student kitchen]. I haven't tried and sometimes I make excuses to not cook." (P20, G5)<br>"I would try to stop stressing out like I know it's hard, but I eat a lot when I get stressed out or when I'm studying…I would like to try to stop myself or try to keep zero snacks or zero food in my room." (P20, G5)

"Next year I plan on living off campus and I can do a better job eating healthy myself rather than relying on the [campus dining center] to give me my choices." (P17, G4)

"[Make better] choices in general, I already stopped eating chips and candy" (P21, G5)

"…limiting myself to how much I can eat of a certain food." (P19, G5)
